# Computer-Aided Detection (CADe) of Small Metastatic Prostate Cancer Lesions on 3D PSMA PET Volumes Using Multi-Angle Maximum Intensity Projections

**DOI:** 10.3390/cancers17091563

**Published:** 2025-05-03

**Authors:** Amirhosein Toosi, Sara Harsini, Ghasemali Divband, François Bénard, Carlos F. Uribe, Felipe Oviedo, Rahul Dodhia, William B. Weeks, Juan M. Lavista Ferres, Arman Rahmim

**Affiliations:** 1 Department of Integrative Oncology, BC Cancer Research Institute, Vancouver, BC V5Z 1L3, Canada; amirhosein.toosi@gmail.com (A.T.); curibe@bccrc.ca (C.F.U.); 2 Department of Radiology, University of British Columbia, Vancouver, BC V6T 1Z3, Canada; fbenard@bccrc.ca; 3 AI for Good Lab, Microsoft Corporation, Redmond, WA 98052, USA; felipe.oviedo@microsoft.com (F.O.); rahul.dodhia@microsoft.com (R.D.); wiweeks@microsoft.com (W.B.W.); jlavista@microsoft.com (J.M.L.F.); 4 BC Cancer, Vancouver, BC V5Z 1L3, Canada; sharsini@bccrc.ca; 5 Nuclear Medicine Center, Jam Hospital, Tehran 1997667665, Iran; divband_ali@yahoo.com; 6 Department of Physics and Astronomy, University of British Columbia, Vancouver, BC V6T 1Z3, Canada

**Keywords:** prostate-specific membrane antigen (PSMA) PET, prostate cancer, computer-aided detection, lesion detection, deep learning, segment anything

## Abstract

Objectives: We aimed to develop and evaluate a novel computer-aided detection (CADe) approach for identifying small metastatic biochemically recurrent (BCR) prostate cancer (PCa) lesions on PSMA-PET images, utilizing multi-angle Maximum Intensity Projections (MA-MIPs) and state-of-the-art (SOTA) object detection algorithms. Methods: We fine-tuned and evaluated 16 SOTA object detection algorithms (selected across four main categories of model types) applied to MA-MIPs as extracted from rotated 3D PSMA-PET volumes. Predicted 2D bounding boxes were back-projected to the original 3D space using the Ordered Subset Expectation Maximization (OSEM) algorithm. A fine-tuned Medical Segment-Anything Model (MedSAM) was then also used to segment the identified lesions within the bounding boxes. Results: The proposed method achieved a high detection performance for this difficult task, with the FreeAnchor model reaching an F1-score of 0.69 and a recall of 0.74. It outperformed several 3D methods in efficiency while maintaining comparable accuracy. Strong recall rates were observed for clinically relevant areas, such as local relapses (0.82) and bone metastases (0.80). Conclusion: Our fully automated CADe tool shows promise in assisting physicians as a “second reader” for detecting small metastatic BCR PCa lesions on PSMA-PET images. By leveraging the strength and computational efficiency of 2D models while preserving 3D spatial information of the PSMA-PET volume, the proposed approach has the potential to improve detectability and reduce workload in cancer diagnosis and management.

## Simple Summary

We aimed to develop an automated computer-aided detection (CADe) system to help doctors detect small metastatic prostate cancer (PCa) lesions more efficiently, ultimately acting as a “second reader” to improve diagnosis and reduce workload in cancer care. Our method used multi-angle Maximum Intensity Projections (MA-MIPs) and explored state-of-the-art (SOTA) object detection AI algorithms. We evaluated 16 SOTA models across four categories. The system identified lesions in 2D images and then mapped them back into 3D space. A fine-tuned segmentation model further refined the results. Our best model, FreeAnchor, achieved a stronger detection performance. It was more efficient than many 3D methods while maintaining high accuracy, and it performed especially well for local relapses and bone metastases.

## 1. Introduction

Prostate cancer (PCa) is the second most common type of cancer and the fifth leading cause of cancer-related death in men worldwide [1]. Despite the advancements in PCa-localized treatments such as radical prostatectomy (RP) or radiation therapy (RT), nearly one-third of patients will encounter biochemical recurrent disease (BCR) [2]. Biochemical recurrence, indicated by an increase in prostate-specific antigen (PSA) levels in blood [3], may manifest as metastasis in the local lymph nodes and/or bone structures in its early stages. With the advancement of disease, distant organs such as the liver and lungs [4] may also be affected, and depending on the involved anatomical sites, different types of treatments may be required. Hence, accurately locating the areas of disease recurrence is crucial for therapeutic decision-making. Although the elevated PSA level in the blood is proven to be the main indicator for the disease recurrence in PCa patients [5], it does not grant the ability to localize the reappearance site of the disease. Therefore, it is essential to use a diagnostic imaging modality with high sensitivity and specificity to localize the disease, distinguish between different types of disease (local relapse, oligometastatic disease, or extensive disease), and ultimately perform personalized treatment planning for the patients.

Prostate-specific membrane antigen (PSMA), as a powerful target for positron emission tomography (PET) imaging, is revolutionizing the diagnosis and management of PCa [5]. The development of new radiopharmaceuticals able to target the PSMA protein, e.g., [^68^Ga]Ga-PSMA-11, [^18^F]PSMA-1007, and [^18^F]DCFPyL, has significantly contributed to improved PCa diagnosis, treatment, decision-making, and patient care management [6]. This has enabled a theranostic paradigm in PCa where PSMA theranostic pairs can be used to image and treat PCa.

Deep learning-based models, particularly those leveraging convolutional neural networks and, more recently, transformers, have demonstrated promising potential in computer-aided detection (CADe) and diagnosis (CADx) [7]. Nevertheless, they encounter numerous challenges intrinsic to biomedical image modalities, including the 3D nature of image modalities; diffused, irregular, and unclear boundaries between different regions; lack of color information; limited contrast; low signal-to-noise ratio, especially in low-dose image acquisition protocols; motion blur; and significant intra- and inter-patient variabilities [8,9,10]. Moreover, unique aspects of PSMA-PET imaging add to these complications, including low spatial resolutions (leading to partial volume effects), high levels of noise, reconstruction artifacts, and limited anatomical information [11].

Furthermore, specific characteristics associated with BCR PCa further complicate image interpretation even for experienced physicians, including the presence of local or distant metastatic lesions with very small sizes and very low radiopharmaceutical uptakes with a high probability of being located at a near proximity to organs with high physiological uptakes such as the urinary bladder, ureters, and kidneys. These specific factors make the process of manual segmentation for the BCR PCa lesions time-consuming and labor-intensive. As a result, the computer-aided detection (CADe) of BCR PCa lesions prior to performing manual or automatic segmentation could enhance the efficiency of these processes and potentially reduce physician workload and increase overall diagnostic precision.

Subsequently, the present study introduces a novel approach to detecting small metastatic lesions on PSMA-PET images. By leveraging state-of-the-art 2D object detection algorithms on multiple Maximum Intensity Projections (MIPs) computed from different angles around the 3D PSMA-PET volumes, i.e., multi-angle MIPs (MA-MIPs), our proposed method overcomes the limitations of conventional 2D, 2.5D, and 3D segmentation methods in detecting very small and low-PSMA-uptake metastatic PCa lesions. The proposed four-step framework, consisting of MA-MIP generation, 2D detection, 3D back-projection, and SAM-based segmentation for small metastatic lesions, offers a very promising pathway to enhance the accuracy and efficiency of PCa lesion detection in clinical practice and improve the early detection and localization of BCR disease. It has great potential to assist physicians, acting as a “second reader” to improve detection sensitivity. Our proposed method takes advantage of the computational efficiency and performance benefits of state-of-the-art 2D object detection models on MA-MIPs, without losing the spatial information of the 3D volume. We show that our method outperforms its state-of-the-art 3D segmentation rivals in terms of various lesion-based, voxel-based, and patient-based detection and segmentation metrics on the target dataset.

The rest of this article presents our proposed methodology for PCa lesion detection and segmentation, including data preprocessing and MA-MIP generation (Section 2.1 and Section 2.2), object detection model fine-tuning (Section 2.3), 3D boxes back-projection (Section 2.5), and SAM-based segmentation (Section 2.6), followed by results (Section 3) and discussion of implications (Section 4).

## 2. Materials and Methods

### 2.1. Dataset Specifications

This is a post hoc sub-group analysis of a prospective investigator-initiated clinical trial. The inclusion criteria were the following:

1. Histologically proven PCa with BCR after initial curative therapy with radical prostatectomy, with a PSA > 0.4 ng/mL and an additional PSA measurement confirming an increase.
2. Histologically proven PCa with BCR after initial curative radiotherapy, with a PSA > 2 ng/mL after therapy [12].

**Figure 1:**
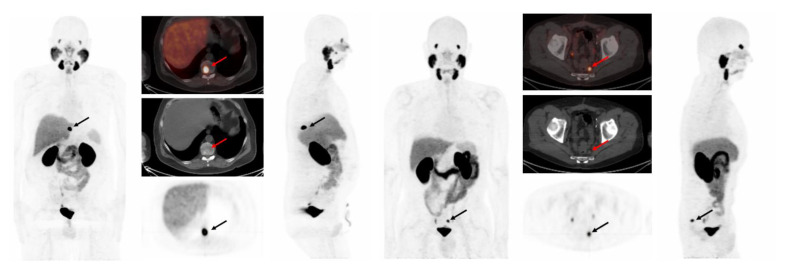
(**Left**) A 72-year-old man with prostate cancer (pT3a, pN0, Gleason score 9 [5 + 4]) status post-radical prostatectomy in 2015, presented for biochemical recurrence evaluation with PSA rising to 4.6 ng/mL. [^18^F]DCFPyL PET/CT revealed a single lesion with PSMA uptake in the T10 vertebra (SUVmax 36.53), consistent with bone metastasis. (**Right**) A 71-year-old man with prostate cancer (pT3b pN0, Gleason score 9 [4 + 5]) status post-radical prostatectomy in 2019, presented for biochemical recurrence evaluation with PSA rising to 2.3 ng/mL. [^18^F]DCFPyL PET/CT detected a presacral lymph node with PSMA uptake (SUVmax 12.3). Both PSMA-avid lesions were successfully detected by all 16 proposed detection methods investigated in this work.

Overall, 317 whole-body [^18^F]DCFPyL PSMA-PET/CT images from the same number of patients were chosen, specifically being consecutive oligometastatic cases having up to five lesions (see Table 1 for patients and tumors characteristics and Figure 1 for examples of patient images). All PET scans occurred after primary treatment, which could have been radiation therapy, prostatectomy, or both. The time from initial therapy to PET/CT had a median (range) of 7 (1–20) years. This was a single-center study, conducted at BC Cancer. After a 4 h fast, participants were injected intravenously with 237–474 MBq of [^18^F]DCFPyL (scaled by body weight), allowing for a 10% variation in target activity. At 120 min post-injection, patients were imaged from the vertex to the mid-thigh using a Discovery PET/CT 600 or 690 scanner (GE Healthcare). A non-contrast-enhanced CT scan for localization and attenuation correction (120 kV, automatic mA selection [30–200 mA range], noise index 20) was acquired, followed by a PET scan (2–4 min/bed position) reconstructed with OSEM and point-spread function modeling. Each trans-axial PET image had a matrix size of 192 × 192 pixels, with each pixel covering (3.64 mm)^2^ in physical space.

Manual segmentation was performed on all active lesions by an expert nuclear medicine physician. On average, each image had 1.92 ± 1.21 PCa lesions with an average active volume of 4.02 ± 9.74 mL and a long axis diameter of 12.96 ± 10.11 mm (on CT). A total of 606 lesions were segmented, where the average maximum and mean standard uptake values (SUVmax and SUVmean) over all the lesions were 7.28 ± 10.36 and 3.62 ± 3.64, respectively. Detailed characteristics of the dataset are provided in Table 1.

### 2.2. Data Preprocessing

**Figure 2:**
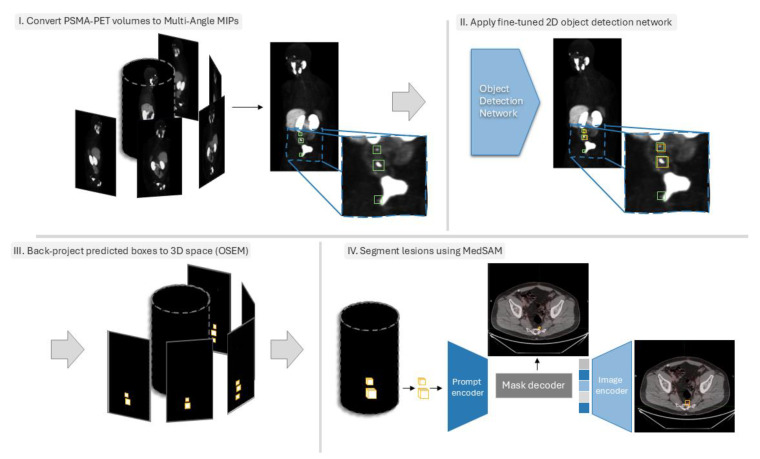
Overview of the proposed method. (**I**). First, the 3D PSMA PET volume is converted to MA-MIPs, and the segmentation masks of the lesions are also converted to bounding boxes. (**II**). A set of pre-trained 2D object detection networks are fine-tuned on the MA-MIP images and then used to predict the location of lesions on MA-MIPs of the test samples. (**III**). Predicted bounding boxes are then converted into binary masks and back-projected to the original 3D space using the OSEM framework. (**IV**). The 3D bounding boxes are finally used as prompts for fine-tuned MedSAM to segment the lesions.

PSMA-PET activity concentration values (Bq/mL) were converted to standard uptake value (SUV). To decrease the contrast between normal organs with high physiological uptake values and the small-sized lesions with relatively lower uptakes, SUVs of the 3D volumes were clipped to a range of 0 and 10. This clipping could cause saturation in the uptake values of high-uptake normal organs but at the same time helps the small lesions, especially with very low SUVs, to be more visible in contrast with the background. Subsequently, SUV images were normalized to zero mean and unit variance, then transformed into the range of zero and one. Trans-axial PSMA-PET slices were then up-sampled from their original matrix size (192 × 192) to the CT matrix size (512 × 512) using third-order bilinear interpolation. Next, 35 axial rotations of the 3D PSMA-PET volumes were computed in every 10 degrees of rotation, and MIPs of all 36 volumes were computed (see Figure 2). The same transformations were applied to the 3D binary segmentation mask volume, except for resampling, where nearest-neighbor interpolation was employed to preserve the sharp boundaries.

To leverage the pre-trained object detection models trained on large image datasets (e.g., MS COCO, PASCAL VOC, etc.) and fine-tune them on our target dataset, we convert the 2D single-channel gray-scale MA-MIP images to 3-channel inputs by duplicating them to the remaining 2 channels. Subsequently, inscribed bounding boxes for all projected segmentation masks were computed. In 2D MA-MIPs, it is common to encounter the partial or full occlusion of some lesions by high-uptake normal organs, especially for locally relapsed lesions in the near proximity of physiologically high-uptake normal organs such as the bladder, where the presence of lesions is highly probable. Therefore, to avoid training models with misleading annotations, the resulting bounding boxes generated from the projection of manual delineations were refined (modified or removed in partial- or full-occlusion situations, respectively). Bounding box annotations were then converted to the proper standard (e.g., PASCAL VOC, MS COCO, YOLO, etc.) for fine-tuning the object detection models, depending on the implementation of the network.

### 2.3. Object Detection Networks Training

Given the small sizes of recurrent PCa lesions, in accordance with the definitions of object sizes in standard object detection evaluation metrics provided by Microsoft Common Objects in Context (COCO) [13], we targeted (among state-of-the-art object detection methods in the literature) ones with higher performances, particularly in detecting small objects. As a result, we picked 16 state-of-the-art (SOTA) architectures available with pre-trained weights with the highest mean average precision (mAP) on small objects to assess their ability to detect PCa lesions on PSMA-PET MA-MIP images. Table 2 summarizes the selected models for this study. Network architecture was selected in an attempt to span methods from all the available sub-categories of object detection models in the literature, namely multi-stage, single-stage, anchor-free, and query-based methods.

We fine-tuned the 16 SOTA object detection networks (Table 2) from four main categories of model types on all 36 MA-MIPs of 267 PSMA-PET volumes of the patients in the training set. For each network, we started fine-tuning from the pre-trained model for 12 epochs using the schedules originally used for training the models. Early-stopping was used based on the mean average precision (mAP) of the bounding boxes on the validation set. Our dataset was randomly split into 287 images for training and validation and 30 unseen images for testing the fine-tuned models.

### 2.4. Back-Projecting Predicted Bounding Boxes Using OSEM

The Ordered Subset Expectation Maximization (OSEM) is an iterative 3D image reconstruction algorithm widely used for tomographic imaging modalities such as positron emission tomography (PET) and Single-Photon Emission Computed Tomography (SPECT) [30]. OSEM reconstructs 3D images from a series of 2D tomographic projections acquired at different angles around the patient’s body.

In this work, we employed, for the first time to our knowledge, the OSEM framework to back-project the predicted bounding boxes of the lesions from 2D MA-MIP space onto the 3D volume space. To this end, we first converted the predicted bounding boxes to 2D binary masks, defining the background pixels as zero and the area inside the bounding boxes as one. Subsequently, similar to image reconstruction, we applied OSEM to the binary masks to back-project the 2D bounding boxes to the 3D volume space.

Let $V\;:\;{\mathbb{R}}^{3}⟶\mathbb{R}$ be the 3D PET volume for a sample patient, where $V\left(x,y,z\right)$ represents the intensity at position $\left(x,y,z\right)$. For any given angle $\theta \in \left[0,2\pi \right)$, the Maximum Intensity Projection (MIP) is defined as follows:

(1) $$MIP_{\theta }\left(u,v\right)=max\left\{V\left(x,y,z\right)\;|\;x\;cos\theta +y\;sin\theta =u,-x\;sin\theta +y\;cos\theta =v,z\in \mathbb{R}\right\}$$

where $\left(u,v\right)$ are coordinates in the projection plane. Now, let $B_{\theta }=\left\{\left(u_{1},v_{1},u_{2},v_{2}\right)\;|\;u_{1}<u_{2},v_{1}<v_{2}\right\}$ be the set of bounding boxes detected on $MIP_{\theta }$. For each bounding box $b\in B_{\theta }$, we define the binary mask $M_{\theta ,b}\;:\;{\mathbb{R}}^{2}\;⟶\left\{0,1\right\}$ as follows:

(2) $$M_{\theta ,b}\left(u,v\right)=\left\{\begin{array}{ll} 1, & if\;\left(\;u_{1}\leq \;u\;\leq u_{2}\right)\;and\;\left(\;v_{1}\leq \;v\;\leq v_{2}\right)\\ 0, & otherwise \end{array}\right.$$

Let $f:\;{\mathbb{R}}^{3}⟶\mathbb{R}$ be the reconstructed 3D volume. The back-projection operation (invoked within the OSEM framework) can be expressed using a modified inverse Radon transform:

(3) $$f\left(x,y,z\right)=\frac{1}{N}\overset{N-1}{\underset{i=0}{\sum }}M_{\theta_{i}}\left(x\;cos\theta_{i}+y\;sin\theta_{i}\right)$$

where *N* is the number of projections, and $\theta_{i}=\frac{2\pi i}{N}$. Then, the OSEM reconstructed volume can be thresholded to create a binary 3D shape:

(4) $$C\left(x,y,z\right)=\left\{\begin{array}{ll} 1, & \;if\;f\left(x,y,z\right)\;\geq \;T\\ 0, & \;otherwise \end{array}\right.$$

where *T* is a chosen threshold value based on the validation subset.

Each binary mask $M_{\theta }$, when simply back-projected, represents a rectangular prism (cuboid) in 3D space, extending in the direction perpendicular to the projection plane. As we rotate through all angles, these prisms intersect. The intersection of all these prisms ideally forms an elliptic cylinder. The dimension of a 3D cuboid circumscribing an elliptic cylinder will be 2a×2b×h, where a denotes the semi-major axis of the ellipse, b denotes the semi-minor axis of the ellipse, and h is the height of the cylinder. The 3D cuboids can then be used as a prompt for performing the automatic segmentation of the lesions inscribed in the cuboids, thanks to promptable universal segmentation foundation models like SAM.

### 2.5. Lesion Segmentation Within 3D Bounding Boxes Using MedSAM

To perform the automatic segmentation of the lesions inscribed in the 3D cuboids, we used MedSAM [31]. MedSAM is a vision foundation model for medical image segmentation based on the SAM architecture. MedSAM is trained on a large-scale biomedical image dataset comprising 1.5M image-mask pairs, from 10 different modalities and 30 cancer types. However, PET imaging modalities are not among the data modalities used for training MedSAM. As a result, we further fine-tuned only the image decoder of MedSAM for a few epochs on our dataset, while keeping both the image and the prompt encoder of the architecture frozen while fine-tuning. Like SAM, MedSAM is a promptable 2D segmentation foundation model that requires point or bounding boxes to specify the target object. As a result, during training, in addition to 3D PSMA PET CT volumes as a series of axial 2D slices, the 3D cuboids are fed to the model as rectangular cross-sections of the 3D bounding cuboid for each axial slice. The performance of the proposed MA-MIP detection framework was evaluated using multiple complementary metrics, including lesion-based detection metrics and tissue-specific performance analysis, as well as segmentation metrics.

### 2.6. Experimental Details

The code for training and evaluating the object detection models and back-projection of 2D MA-MIPs to 3D volume space was implemented in Python 3.8, using Pytorch 1.9, CUDA 11.3, Open-MM Lab’s mmDetection framework [32], and the PyTomography toolbox. Fine-tuning the code for MedSAM was performed on the same training data as the detection models and was implemented using Python 3.10, Pytorch 2.3, and CUDA 12.8. Our proposed techniques were additionally compared to multiple fine-tuned conventional 3D segmentation methods (Table 3), including AttentionUNet, FlexibleUNet, SegResNet, SwinUNETR, UNet, UNetPlusPlus, VNet, UNETR, and nnUNet. The training and evaluation of the models were performed on two instances of Microsoft Azure Ubuntu VMs (16.04 and 18.04 Lts.), each equipped with four NVIDIA Tesla V-100 16GB GPUs. All results shown are for the n = 30 unseen test cases (hold-out subset of the same set used for training and validation).

## 3. Results

In terms of overall detection performance in this difficult task (see Table 3), among the 16 implemented MA-MIP detection approaches, FreeAnchor demonstrated superior overall performance with the highest F1-score (0.69) and recall (0.74), while maintaining a reasonable precision of 0.65. This was followed by Deformable DETR and Sparse R-CNN, achieving F1-scores of 0.67 and 0.65, respectively. The highest precision was achieved by ATSS (0.86), though at the cost of a substantially lower recall (0.33). Multi-stage detectors showed consistently robust performance, with Cascade R-CNN and Trident-Net both achieving high recall (0.70) while maintaining moderate precision (0.55 and 0.53, respectively). This aligns with our primary objective of maximizing true-positive detections while maintaining acceptable false-positive rates. When compared to conventional 3D segmentation approaches (as elaborated in Table 3), our MA-MIP detection framework showed competitive performance. While nnUNet achieved the highest F1-score (0.72) among all methods, many 3D segmentation models suffered from excessive false positives, as evidenced by precision scores as low as 0.15 (UNETR) and 0.17 (AttentionUnet).

**Figure 3:**
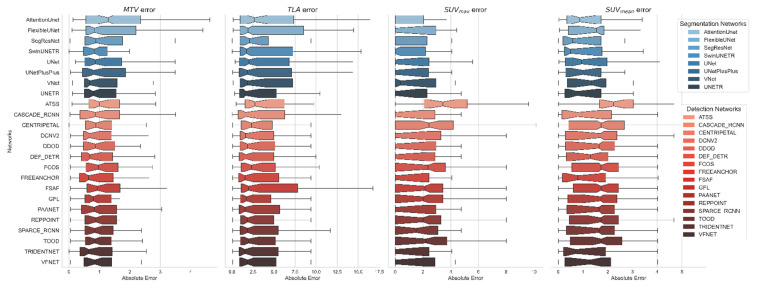
Error distributions for 4 clinical biomarkers (MTV, TLA, SUVmean, and SUVmax) computed based on the predicted segmentation masks of all 3D segmentation models (blue tone plots; top) and proposed MA-MIP detection-based models (red tone plots; bottom).

An analysis of tissue-specific detection performance (see Table 4) revealed distinct patterns across different anatomical locations. All methods demonstrated strong performance in detecting local relapses, with recall rates consistently above 0.70. PAA Net and nnDetection achieved the highest recall (0.91) for local relapse detection, followed closely by several other methods, including FreeAnchor and Cascade R-CNN (0.82). Regional lymph node detection showed moderate performance across all methods, with FreeAnchor and Cascade R-CNN achieving the highest recall (0.70). The detection of distant lymph nodes proved more challenging, with recall rates generally lower than those for regional nodes. Several methods, including Cascade R-CNN, DCNv2, and Trident-Net, achieved 0.67 recall for distant lymph nodes. Performance in bone metastasis detection varied considerably across methods. FreeAnchor and Varifocal-Net achieved the highest recall (0.80), while several methods struggled with recall rates below 0.50. This variability likely reflects the diverse presentation and challenging nature of bone metastases in PSMA-PET imaging. An analysis of clinical biomarkers (MTV, TLA, SUVmean, and SUVmax) revealed interesting patterns in error distributions between MA-MIP detection-based and 3D segmentation approaches (Figure 3). MA-MIP detection methods generally showed more consistent error distributions across all biomarkers compared to 3D segmentation methods, which exhibited greater variability.

**Figure 4:**
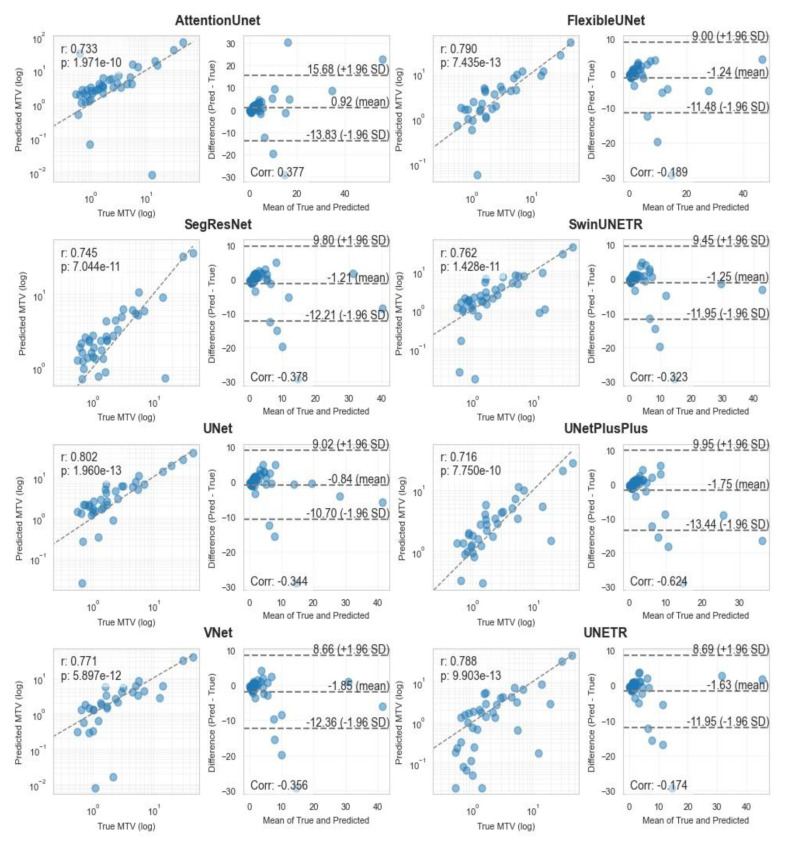
Pearson correlation and Bland–Altman diagrams of MTV biomarker, calculated using all 8 3D segmentation methods.

**Figure 5:**
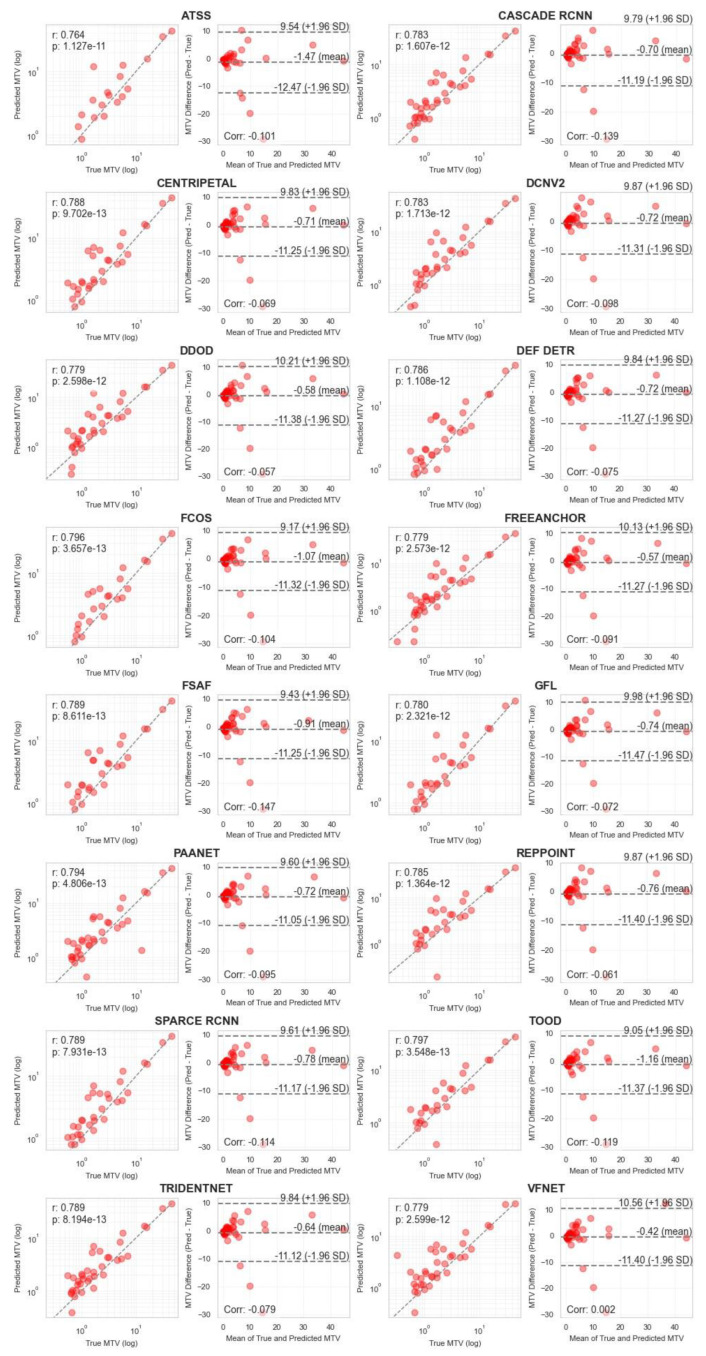
Pearson correlation and Bland–Altman plots of MTV biomarker, calculated using all 16 MA-MIP detection methods.

Correlation analysis for MTV measurements (Figure 4 and Figure 5) demonstrated strong agreement between ground truth and predicted values across both methodologies. MA-MIP detection methods showed particularly strong correlation coefficients, with Bland–Altman plots revealing consistent performance across the range of MTV values. When considering segmentation quality metrics (Table 5), Deformable DETR achieved the lowest volumetric error (39.4%) among MA-MIP detection methods, while maintaining competitive Dice scores (0.47) and sensitivity (0.35). This performance was comparable to the best-performing 3D segmentation method, SwinUNETR (volumetric error: 40.64%, Dice: 0.46). These results demonstrate that our proposed MA-MIP detection framework can achieve comparable or superior performance to conventional 3D approaches while maintaining better computational efficiency and requiring fewer complex annotations for training.

## 4. Discussion

BCR develops in almost one-third of men with PCa after local therapy [2]. There are multiple options for subsequent management, and PSMA PET imaging has a crucial role in this setting, with increasing efforts to promote the accurate detection of BCR in earlier stages by PSMA PET [12]. Despite the increased use of AI in biomedical imaging, applications to PCa metastatic lesion segmentation remain limited. Existing research has predominantly focused on local primary (intra-prostatic) tumor segmentation [33], a comparatively less complex task. Only a small number of studies have explored the use of AI segmentation models for PCa metastatic lesions [34,35]. Notably, while Refs. [34,35] evaluated such models, only the latter (from our team) utilized a dataset specifically comprising PCa recurrence patients. This underscores the need for further research in this area, particularly in the context of metastatic disease.

In the current study, a CADe method for localizing small metastatic BCR PCa lesions was developed by coupling MA-MIPs with deep learning-based object detection algorithms, with promising results. It showed the strength of the proposed method in identifying distant metastases, both in terms of distant involved lymph nodes and bone metastatic lesions, while maintaining comparable performance in detecting local relapse and regional lymph node involvement. The proposed method achieved a higher detection rate (e.g., FreeAnchor: F1-score: 0.69, recall: 0.74) while generating fewer false positives compared to conventional 3D-based segmentation approaches (e.g., FreeAnchor: #FP = 22 vs. SegResNet: #FP = 160 and Attention UNet: #FP = 193), addressing a critical clinical need in PCa management.

The higher performance in detecting distant metastases, particularly bone metastases (FreeAnchor—recall: 0.80), has significant clinical implications. The ability to accurately identify and localize distant metastases is crucial for treatment planning and prognosis assessment. Interestingly, these higher performances were achieved without leveraging the anatomical information of CT images, suggesting that the MA-MIP-based approach can be sensitive to subtle uptake patterns that might be missed in conventional analysis based on 3D volumetric PET/CT images.

The proposed method provides a few advantages over existing conventional 3D-based methods. Training the networks on 2D MA-MIPs instead of full 3D volumetric data requires fewer computational and memory resources while maintaining higher-resolution input image processing capabilities, letting models process the entire spatial information instead of lowering the 3D resolution of the input images or leveraging patch-based methods. Our framework also provides the benefit of leveraging valuable pre-trained 2D object detection models with knowledge learnt from the rich background of natural images, while avoiding the expenses of training 3D models. In addition, the proposed MA-MIP-based method demonstrates more control over false positives compared to the conventional 3D methods, potentially removing the need for further manual verification or lowering the cost in terms of required time and labor.

There are a number of limitations to the proposed methodology. The current lack of incorporating CT information through MIPs represents a limitation, potentially affecting the detection of lesions with a more dominant presence on a CT scan. The dataset used in the current study relies on single-expert manual segmentation. Our dataset of 317 whole-body PSMA-PET/CT images is substantial; however, the model’s generalizability to different institutional datasets and patient populations requires further validation. The use of pre-trained models on natural image datasets introduces a domain gap that may not be fully addressed by fine-tuning. Similarly, MedSAM’s lack of initial training on any PET imaging modality may affect segmentation accuracy. The absence of testing on external datasets limits our ability to assess the method’s generalizability across different clinical settings and acquisition protocols. Finally, it should be noted that the performance values reported in this study, particularly for bone metastasis detection, may not directly translate to other PSMA-targeting radiopharmaceuticals, which differ in biodistribution and false positive profiles. As such, this represents a limitation of our study and highlights the need for further validation across different radiopharmaceuticals.

Future research directions include the integration of CT anatomical information through an alternative approach of MIPs able to preserve anatomical context while maintaining similar computational efficiency; multi-center external validation studies to assess the generalizability of the proposed approach across different settings and patient populations; employing domain-specific pre-training methods for both 2D object detection methods and MedSAM segmentation model to better address the domain gap between natural and medical images; and assessing inter-observer variability by means of utilizing datasets annotated by multiple experts.

Overall, our proposed CADe framework takes advantage of the computational efficiency and performance benefits of 2D object detection models on MA-MIPs, without losing the spatial information of the 3D volume. Our results revealed that PCa lesions can be detected more reliably and efficiently compared to more conventional 3D detection methods. The method’s strong performance in detecting distant metastases (distant lymph nodes and bone lesions) suggests potential value as a clinical decision support tool. Enabling CADe as a “second reader” with a fast overview and focus on suspicious lesions can aid reading physicians in performing the more timely and accurate identification and evaluation of lesions. It can also enable the routine quantification of disease burden, such as MTV measurements. It is expected that by increasing the number of samples in subsequent studies and improving training data, this novel framework will be enhanced to accurately detect PCa recurrence even in smaller lesions and/or lower levels of PSA.

## 5. Conclusions

In this study, we developed an efficient CADe framework for detecting small metastatic prostate cancer lesions on PSMA-PET images using MA-MIPs and deep learning. Our approach demonstrated strong detection performance, especially for distant metastases, while maintaining high computational efficiency. By leveraging state-of-the-art 2D object detection models while preserving 3D spatial information, our method outperformed conventional 3D segmentation approaches. This novel framework shows promising potential to assist clinicians in improving detection and treatment planning and improving workflow in prostate cancer care.

## Figures and Tables

**Table 1:** Patient and tumor characteristics in the present study.

Characteristic	Entire Cohort (*N* = 317)
Age at diagnosis (years), median (range)	71 (45–91)
*Initial Gleason score*	
6	31 (9.6%)
3 + 4	81 (25.3%)
4 + 3	108 (33.7%)
8	36 (11.3%)
9	58 (18.1%)
*Primary tumor classification*	
p/cT1	38 (11.9%)
p/cT2	144 (45%)
p/cT3	129 (40.3%)
p/cT4	3 (0.9%)
p/cTx	3 (0.9%)
*Primary nodal status*	
p/cN0	223 (69.7%)
p/cN1	24 (7.5%)
p/cNx	72 (22.5%)
*D’Amico Score*	
High risk	241 (75.31%)
Intermediate risk	63 (19.68%)
Low risk	18 (5.62%)
PSA at PET/CT (ng/mL), median (range)	3.2 (0.06–53.3)
*Number of lesions at PET/CT*	
1	170 (53.1%)
2	66 (20.6%)
3–5	85 (26.6%)
*Site of lesion*	
Local recurrence	162 (50.6%)
Regional lymph node	130 (40.6%)
Distant lymph node	41 (12.8%)
Bone	59 (18.4%)
Other	11 (3.4%)
SUVmax (g/mL), median (range)	7.51 (0.97–97.39)
TMTV (mL), median (range)	4.02 (0.18–92)
TLA (g), median (range)	16.02 (0.35–716.87)

PSA: prostate-specific antigen, SUVmax: maximum standardized uptake value, MTV: metabolic tumor volume, TLA: total lesion activity.

**Table 2:** List of object detection methods used in this work, along with their backbone architecture, their category, and their average precision (AP) values on small-sized objects of COCO dev-test. R-50: ResNet-50, R-101-DCN: ResNet-101 with Deformable Convolution blocks, X-101: Xception-101, H-104: Hourglass-104.

Object Detection (OD) Network	Model Type	Backbone	AP
Cascade R-CNN [14]	Multi-stage	R-50	26.6
Sparse R-CNN [15]	Multi-stage	R-101	28.3
Deformable ConvNets v2 (DCNv2) [16]	Multi-stage	R-101-DCN	27.8
Trident-Net [17]	Multi-stage	R-101-DCN	28
Adaptive Training Sample Selection (ATSS) [18]	Single stage	R-101	26.1
FreeAnchor [19]	Single stage	X-101	26.8
Probabilistic Anchor Assignment with IoU Pred. [20]	Single stage	R-101-DCN	27.9
Generalized Focal Loss (GFL) [21]	Single stage	X-101-DCN	29.2
Varifocal-Net [22]	Single stage	X-101-DCN	30.7
Disentangle Your Dense Object Detector (DDOD) [23]	Single stage	R-50	27.2
Task-aligned One-stage Object Detection (TOOD) [24]	Single stage	R-101-DCN	30.5
RepPoints [25]	Anchor-free	R-101-DCN	30.3
Feature Selective Anchor-Free (FSAF) [26]	Anchor-free	X-101	26.6
Fully Convolutional One-Stage Object Detection [27]	Anchor-free	X-101	27.6
Centripetal Net [28]	Anchor-free	H-104	29
Deformable DETR [29]	Query-based	R-50	27.7

**Table 3:** The detection performance of our applied techniques in terms of detection errors (TP, FP, and FN), precision, recall, and F1-score. TP (true positive): The number of objects that are correctly detected by the model. FP (false positive): The number of objects that are falsely detected by the model. FN (false negative): The number of objects that are missed or not detected by the model. Precision: The percentage of correct detections among all detections made (TP/(TP + FP)). Recall: The percentage of actual objects that were detected (TP/(TP + FN)). F1-score: The harmonic mean of precision and recall (2 × Precision × Recall/(Precision + Recall)) or a balance between precision and recall. Results are shown for the test set (30 patients, 54 lesions). Best performances are shown in bold.

		Networks	TP	FP	FN	Precision	Recall	F1-Score
Detection Models	Multi-Stage	Cascade R-CNN	38	31	16	0.55	0.70	0.62
		Sparse R-CNN	32	12	22	0.73	0.59	0.65
		DCNv2	32	20	22	0.62	0.59	0.60
		Trident-Net	38	34	16	0.53	0.70	0.60
	Single-Stage	ATSS	18	3	36	0.86	0.33	0.48
		FreeAnchor	**40**	22	**14**	0.65	**0.74**	0.69
		PAA Net	33	25	21	0.57	0.61	0.59
		GFL	30	8	24	0.79	0.56	0.65
		Varifocal-Net	35	32	19	0.52	0.65	0.58
		DDOD	35	28	19	0.56	0.65	0.60
		TOOD	25	8	29	0.76	0.46	0.57
	Anchor-Free	RepPoints	29	12	25	0.71	0.54	0.61
		FSAF	25	21	29	0.54	0.46	0.50
		FCOS	25	7	29	0.78	0.46	0.58
		Centripetal Net	23	6	31	0.79	0.43	0.55
	Query-based	Deformable DETR	35	16	19	0.69	0.65	0.67
	3D	nnDetection	38	28	16	0.58	0.70	0.63
Segmentation Models	3D	AttentionUnet	39	193	15	0.17	0.72	0.27
		FlexibleUNet	30	19	24	0.61	0.56	0.58
		SegResNet	39	160	15	0.20	0.72	0.31
		SwinUNETR	36	81	18	0.31	0.67	0.42
		UNet	35	57	19	0.38	0.65	0.48
		UNetPlusPlus	35	68	19	0.34	0.65	0.45
		Vnet	29	19	25	0.60	0.54	0.57
		UNETR	33	193	21	0.15	0.61	0.24
		nnUNet	39	16	15	0.71	0.72	**0.72**

**Table 4:** The tissue-based detection performance of our applied techniques in terms of TP, FN, and recall, along with the overall detection performance for all the lesions throughout the body. Results are shown for the test set (30 patients with 54 lesions, which consisted of 11 lesions as local relapses, 30 regional lymph nodes, 3 distant lymph nodes, and 10 bone metastases (all analyzed), as well as 1 visceral lesion).

			All Lesions					Local Relapse			Regional Lymph Nodes			Distant Lymph Nodes			Bone Metastasis		
		Networks	TP	FP	FN	Recall	F1	TP	FN	Recall	TP	FN	Recall	TP	FN	Recall	TP	FN	Recall
Detection Models	Multi-Stage	Cascade R-CNN	38	31	16	0.70	0.62	9	2	0.82	**21**	**9**	**0.70**	2	1	0.67	6	4	0.60
		Sparse R-CNN	32	12	22	0.59	0.65	8	3	0.73	19	11	0.63	1	2	0.33	5	5	0.50
		DCNv2	32	20	22	0.59	0.60	8	3	0.73	18	12	0.60	2	1	0.67	5	5	0.50
		Trident-Net	38	34	16	0.70	0.60	8	3	0.73	**21**	**9**	**0.70**	2	1	0.67	**8**	**2**	**0.80**
	Single-Stage	ATSS	18	3	36	0.33	0.48	7	4	0.64	8	22	0.27	1	2	0.33	5	5	0.50
		FreeAnchor	**40**	22	**14**	**0.74**	0.69	9	2	0.82	**21**	**9**	**0.70**	2	1	0.67	**8**	**2**	**0.80**
		PAA Net	33	25	21	0.61	0.59	10	1	0.91	20	10	0.67	1	2	0.33	7	3	0.70
		GFL	30	8	24	0.56	0.65	8	3	0.73	18	12	0.60	1	2	0.33	5	5	0.50
		Varifocal-Net	35	32	19	0.65	0.58	9	2	0.82	19	11	0.63	2	1	0.67	**8**	**2**	**0.80**
		DDOD	35	28	19	0.65	0.60	8	3	0.73	19	11	0.63	2	1	0.67	6	4	0.60
		TOOD	25	8	29	0.46	0.57	7	4	0.64	15	15	0.50	2	1	0.67	4	6	0.40
	Anchor-Free	RepPoints	29	12	25	0.54	0.61	8	3	0.73	16	14	0.53	1	2	0.33	4	6	0.40
		FSAF	25	21	29	0.46	0.50	7	4	0.64	16	14	0.53	1	2	0.33	5	5	0.50
		FCOS	25	7	29	0.46	0.58	8	3	0.73	12	18	0.40	1	2	0.33	4	6	0.40
		Centripetal Net	23	6	31	0.43	0.55	8	3	0.73	16	14	0.53	1	2	0.33	6	4	0.60
	Query-based	Deformable DETR	35	16	19	0.61	0.59	8	3	0.73	18	12	0.60	1	2	0.33	6	4	0.60
	3D	nnDetection	38	28	16	0.70	0.63	10	1	0.91	20	10	0.67	2	1	0.67	6	4	0.60
Segmentation Models	3D	AttentionUnet	39	193	15	0.72	0.27	10	1	0.91	**21**	**9**	**0.70**	2	1	0.67	6	4	0.60
		FlexibleUNet	30	19	24	0.56	0.58	9	2	0.82	15	15	0.50	1	2	0.33	5	5	0.50
		SegResNet	39	160	15	0.72	0.31	9	2	0.82	**21**	**9**	**0.70**	2	1	0.67	7	3	0.70
		SwinUNETR	36	81	18	0.67	0.42	**11**	**0**	**1.00**	19	11	0.63	1	2	0.33	5	5	0.50
		UNet	35	57	19	0.65	0.48	9	2	0.82	19	11	0.63	0	3	0.00	6	4	0.60
		UNetPlusPlus	35	68	19	0.65	0.45	9	2	0.82	19	11	0.63	0	3	0.00	6	4	0.60
		Vnet	29	19	25	0.54	0.57	9	2	0.82	14	16	0.47	0	3	0.00	6	4	0.60
		UNETR	33	193	21	0.61	0.24	10	1	0.91	16	14	0.53	0	3	0.00	6	4	0.60
		nnUNet	39	16	15	0.72	**0.72**	9	2	0.82	20	10	0.67	2	1	0.67	7	3	0.70

**Table 5:** The segmentation performance of our applied techniques in terms of Dice metric, 95-percentile Hausdorff Distance, Volume Error, and (voxel-wise) Sensitivity.

		Networks	Dice↑	HD95↓	Vol Err.↓	Sens.↑	
Detection Models	Multi-Stage	Cascade R-CNN	0.45	29.68	47.72	0.33	MedSAM Segmentation model
		Sparse R-CNN	**0.48**	19.42	48.27	**0.35**	
		DCNv2	0.42	21.11	47.19	0.30	
		Trident-Net	0.45	20.34	50.66	0.33	
	Single-Stage	ATSS	0.29	36.81	65.05	0.22	
		FreeAnchor	0.45	24.24	47.98	0.32	
		PAA Net	0.43	24.26	45.58	0.32	
		GFL	0.45	20.08	46.91	0.34	
		Varifocal-Net	0.44	33.71	48.34	0.32	
		DDOD	0.44	30.15	47.15	0.32	
		TOOD	0.40	**9.30**	51.19	0.30	
	Anchor-Free	RepPoints	0.41	19.32	52.57	0.31	
		FSAF	0.38	18.20	51.67	0.28	
		FCOS	0.39	17.83	48.90	0.30	
		Centripetal Net	0.38	18.95	57.52	0.29	
	Query-based	Deformable DETR	0.47	20.53	**39.36**	**0.35**	
	3D	nnDetection	0.45	31.23	47.46	0.32	
Segmentation Models	3D	AttentionUnet	0.35	167.24	66.89	0.24	Task-specific Segmentation models
		FlexibleUNet	0.43	23.53	50.28	0.32	
		SegResNet	0.39	94.18	57.05	0.27	
		SwinUNETR	0.46	18.2	40.64	0.34	
		UNet	0.42	56.02	49.16	0.30	
		UNetPlusPlus	0.40	79.21	52.95	0.28	
		Vnet	0.42	30.26	53.62	0.32	
		UNETR	0.36	80.6	49.61	0.26	
		nnUNet	0.47	30.74	49.90	0.35	

Arrows in metric names indicates whether higher or lower values indicate improved performance.

## Data Availability

Restrictions apply to the availability of these data. PSMA PET/CT data were obtained from the BC Cancer Agency and are available [from http://www.bccancer.bc.ca/] with the permission of the BC Cancer Agency.
